# Assessment of the Use and Status of New Drug Information Centers in a Developing Country, Ethiopia: The Case of Public University Hospital Drug Information Centers

**DOI:** 10.1155/2018/3840976

**Published:** 2018-08-26

**Authors:** Ayenew Ashenef, Elham Reshid, Zewdu Yilma, Tadesse Melaku, Tesfahun Chane

**Affiliations:** ^1^School of Pharmacy, College of Health Sciences, Addis Ababa University, P.O. Box 1176, Addis Ababa, Ethiopia; ^2^Drug Information Centre, School of Pharmacy/Tikur Anbessa Specialized Hospital (TASH), College of Health Sciences, Addis Ababa University, P.O. Box 9086, Addis Ababa, Ethiopia; ^3^Department of Pharmacy, College of Health Sciences, Mekelle University, P.O. Box 1871, Mekelle, Ethiopia; ^4^School of Pharmacy, College of Medicine and Health Sciences, Gondar University, P.O. Box 196, Gondar, Ethiopia; ^5^Department of Pharmacy, College of Medical and Health Sciences, Jimma University, P.O. Box 378, Jimma, Ethiopia

## Abstract

**Introduction:**

Drug information center (DIC), in most cases, is part and parcel of pharmacy service established as a unit that deals with offering recent, balanced, truthful facts about drugs to the public, patients, and health care professionals.

**Objective:**

To assess the query receiving and response trends by the drug information centers (DICs) found in main university affiliated teaching health care institutes in Ethiopia.

**Settings:**

The drug information centers located in Mekelle, Gondar, Jimma, Tikur Anbessa Specialized University Hospitals, and St. Peter Public Hospital, Ethiopia.

**Methods:**

It employed analytical, descriptive (cross-sectional), and retrospective methods. The study was performed from June to August, 2015. All the available documented data were gathered with the help of checklist and questionnaire (self-administered).

**Results:**

A total of 439 queries submitted to the DICs during their active service period were included in this study of which 407 were found to be suitable for this assessment. The highest inquiries had come from public hospitals (60 %) from health care workers (95.1 %), out of which pharmacists were the highest (63.57 %) submitters, followed by health care students (12.7 %). The major purposes of query submission were to improve treatment outcome of patients (33.9 %) and then to update the knowledge (25.1 %) although 39.3 % of the queries did not document it. The most common requests concern drug interaction (19.7 %) followed by therapeutic use (17.8 %) and the major pharmacological group is about antimicrobials (23.3 %) followed by antihypertensives (11.4 %). Web sites (31.4 %) were the most highly used references followed by Micromedex (19.0 %).

**Conclusion:**

The assessment of the DICs had shown that it is feasible to establish and develop DIC services in a developing country setting, Ethiopia. The study found that most of the common queries deal with drug interaction, therapeutic use, and general product information (most commonly about antimicrobials).

## 1. Introduction

Drug information (DI) deals with offering advice about medicines and their role in disease management by oral or written communication methods. Queries may come from health care professionals, health care offering institutes, the general public, and patients [1].

Current advancements in medical sciences are creating huge information about drugs and diseases on a daily basis. The advances being made in drug therapy unfortunately create information gap for health professionals and to patients. This might lead to misuse of drugs. Therefore, drug information services are designed to help all in need of evidence based timely critical information. They offer advice on drugs and clinical care thus contributing significantly in alleviating the world wide problem of misuse of drugs [2].

The current role of pharmacists had changed from the traditional product oriented stream towards provider of pharmaceutical care to patients as part of clinical pharmacy service. This helps to fill any gap that may arise by doctors when medicines are prescribed to patients [3]. Thus, pharmacists had sole and unique responsibility in providing DI as part of their professional duties [4]. Hence pharmacists practicing drug information service had to cope with the vast latest information coming from different scientific literature about numerous new drugs and formulations entering to the market [5]. The same applies to physicians in order to acquire recent advancements in the diagnosis and treatment of diseases [6]. As such pharmacists offering drug information in accurate way are in higher demand [7].

Currently management teams in the health care system are recognizing the importance of drug information center existence. Hence physicians and other health care practitioners will not waste their time in reviewing information about drugs. Instead DICs will perform such duty in an efficient and organized way as central units to physicians, nurses, and other staff [6]. Thus, DIC services fill the time and budget gaps lacking by health care practitioners when they have been required to acquire resources about drug information [5]. Drug information service (DIS) is a unit organized with appropriate materials and staff teamed up to give accurate information about medicines. DIS is usually grown to DIC when the resources and staff number enable them to perform more duties such as research and development, involvement in training, owning positions in the hierarchy, and drawing pharmaceutical attention [8].

In the developed world DICs were started and became functional in the 1960s and 1970s. Later, many high and middle income countries had also established DICs. However in Africa only South Africa and Zimbabwe were pioneers in establishing drug information centers. However, nowadays many countries in Africa including Egypt, Ethiopia, Ghana, Eritrea, Kenya, and Uganda had established DICs [9–15]. But studies assessing these DICs whether they are performing best under the developing country (in Sub-Saharan Africa) scenario or not are rare except few like Uganda [16], Zimbabwe [17], Ethiopia [18], and Sudan [19]. Therefore the aim of this study is to add value and narrow the gap of information that exists in this theme.

Ethiopia, in the past, neither had established drug information centers nor has a well-organized system of disseminating drug information. This may compromise the health care system of the country and societal health. Hence, drug information should be instituted in the health care system. Recently after the pharmaceutical education system is revised and inclined to patient oriented duties, drug information centers are established or in the process in many health care institutes of the country. Currently most university affiliated hospitals in Ethiopia have drug information centers. The first established DIC in the country is that of the TASH DIC in May, 2009, as a support from the PEPFAR, CDC/Ethiopia, Twinning Centre, and Howard University. It was envisioned to be a national model DIC and helped the other DICs to be established and become functional as satellites, namely, the Gondar, Jimma, Mekelle, and Haramaya DICs [20]. But nowadays Ethiopian food medicines and health care administration and control authority (EFMHACA) and Pharmaceutical fund supply agency (PFSA) maintain toll-free telephone hotlines for the purpose drug information services that are tailored to serving the general public [21].

However, the level of awareness to the services, availability of proper resources, and staffing of the centers by well-trained professionals affect the services. In this assessment DICs of the following institutes were included: Tikur Anbessa Specialized Hospital (TASH), Jimma University Teaching Hospital, Gondar University Hospital, Mekelle University Hospital (Ayder Referral Hospital), and St. Peter Public Hospital.

Tikur Anbessa Specialized Hospital is the highest level referral hospital in Ethiopia. The College of Health Sciences of Addis Ababa University is housed in its premise. It is the country's top health care institute with regard to facilities and manpower. It is a 700-bed hospital and had specialized clinical services that are not available in other public or private hospitals in Ethiopia [22].

Jimma University Teaching Hospital (JUTH) is a teaching and referral hospital that serves 15 million people. It is found in Jimma city, 352 km from the capital, in south west Ethiopia. In 2015 the federal government constructed 600-bed facilities which are currently fully functional [23].

The Gondar University Referral Teaching Hospital at Gondar had 400 beds. There are expansion works to the hospital to accommodate state-of-the-art TB ward and laboratory, eye care, fistula service, MCH ward, and general health care. The College of Medicine and Health Sciences (CMHS) of Gondar University located in this hospital offers services to nearby five districts of Gondar and also runs a research center named Dabat research center for public health research activities [24].

Mekelle University Teaching Hospital named the Ayder Referral Hospital is located at Mekelle, Tigray. It became functional in 2008 and serves 8 million populations in the region and its environs. The inpatient bed number is about 500 and the College of Health Sciences of the University is located in this campus [25].

St. Peter Hospital, established in 1960, is a specialized hospital located in Addis Ababa focusing on the treatment of TB patients. It handles drug resistance cases besides other general medical services [26].

### 1.1. Aim of the Study

The objective of this research is to analyze the use and status of DICs located in the four pioneer university teaching hospitals in Ethiopia. Queries and their answers were assessed.

## 2. Methods

### 2.1. Study Place

The assessment was carried out in Addis Ababa, Jimma, Gondar, and Mekelle of Ethiopia. The pioneer university teaching hospitals in the country are included. The hospitals included are the DICs of the Tikur Anbessa Specialized Hospital (TASH), St. Peter Hospital from Addis Ababa, and Capital City of Ethiopia at a central location; Jimma University Teaching Hospital in South West Ethiopia; Gondar University Teaching Hospital in North West Ethiopia; Mekelle University Teaching Hospital (Ayder Referral Hospital) in North Ethiopia.

### 2.2. Research Design

It involves analytical, descriptive (cross-sectional), and retrospective review of queries in/around June, 2015, to August, 2015.

### 2.3. Data Collection and Management

Queries submitted to the DICs were reviewed retrospectively during the study period using a checklist. For TASH queries documented after the previous study [18] were included. The checklist used contains nine questions, which were filled carefully. In addition, a semistructured self-administered questionnaire (containing 17 questions) was filled by the directors of the DICs that had participated in the study.

In summary, the checklist is designed to capture information about enquirer background, patient information, the facility the queries come from, nature of query, how the query was addressed (resources used and reply mechanism), time elapsed to respond, and aim of the submitted question. Similarly, the questionnaire gathers information about the centers professional personnel composition, resources available, duty hours, contact methods, organizational frame, challenges, and working methods. The form was filled by five pharmacists.

Data collected in the study was entered to EPI Info version 3.5.1 (Center for Disease Control and Prevention (CDC), Atlanta, Georgia). SPSS Version 20 (IBM, Chicago, Illinois) was employed for data analysis.

## 3. Results

### 3.1. Number and Distribution of Queries by Source

The number of queries submitted to the DICs totals 439 (Table 1). From such 407 were found to be eligible for the assessment as they contain most of the parameters to be included for this study. Most eligible queries came from Mekelle DIC (35.5 %) with 10.2 queries per month. TASH also received 5.3 queries per month. All inquiries which are documented from the DICs of TASH, Jimma, Mekelle, and St. Peter Hospital were eligible for analysis.

**Figure 1:**
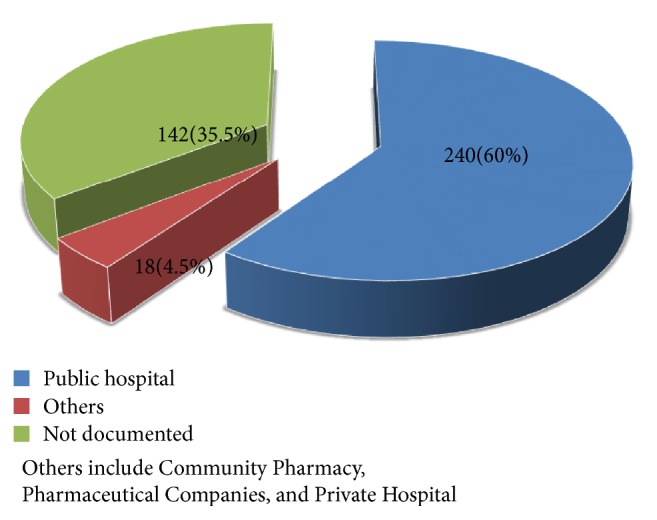
Organizations that sent queries to the drug information centers in hospitals studied.

**Figure 2:**
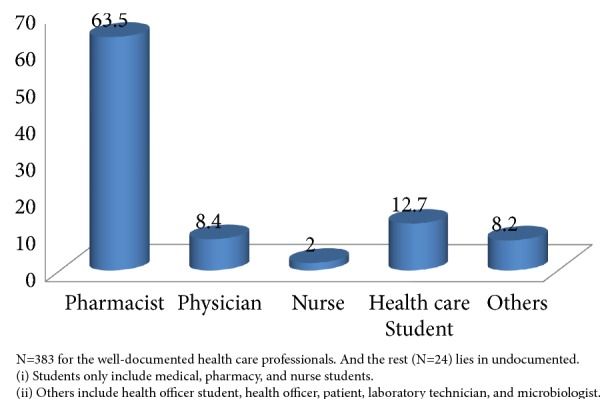
Percentage of health care professionals who submitted queries to the drug information centers in this study.

Most of the requests were from health care professionals (n=385, 99.5 %) (Table 1 and Figure 1), out of which, pharmacists were the highest (63.5 %) followed by health care students (12.7 %), physicians (8.4 %), others (8.2 %), and nurses (2 %) (Figure 2). Queries from the public were very low accounting only for 0.5 % (Table 1 and Figure 1).

### 3.2. Method of Submission of Queries

Health care professionals submitted most of the queries by in person walk-in to the center (45.4 %); the next highest mode is morning round (9.8 %) although significant amount of queries (n=151, 37.8 %) still did not document the mode of receiving. Phone and others (Email and DIC web sites), respectively, account for 5 and 2 % of the mode of receiving the queries (Table 1).

### 3.3. Types and Purpose of Queries

Most of the queries did not document purpose (39.3 %); however better patient care, update to knowledge, and educational as well as academic goals account for 33.9 %, 25.1 %, and 0.5 % of the purposes. The highest number of questions dealt with drug interaction (19.7 %), therapeutic use (17.8 %), and then general product information (13.7 %) (Table 2).

### 3.4. Pharmacological Classification of the Drugs That Come from Inquiries

It deals with antibiotics (23.3 %) followed by antihypertensives (11.4 %), antipains (8.6 %), antihelminths (6.5 %), and antiretrovirals (5.2 %) (Table 3).

### 3.5. Characteristics of Replies

Web sites (31.4 %) were the most common resources used in responding followed by Micromedex (19.0 %). Other most commonly used resources for answering queries were textbooks (15.4 %), UpToDate™ (15.5 %), and journals (14.3 %). With regard to mode of reply, while (n=50) 12. 3 % of the queries are not documented, 323 queries were responded in written form; among them, 188 included rephrased print out documents. In the other (n=45) queries, the mode of reply was verbal employing phone (n=32) and in person (n=13) ways (Table 4).

**Figure 3:**
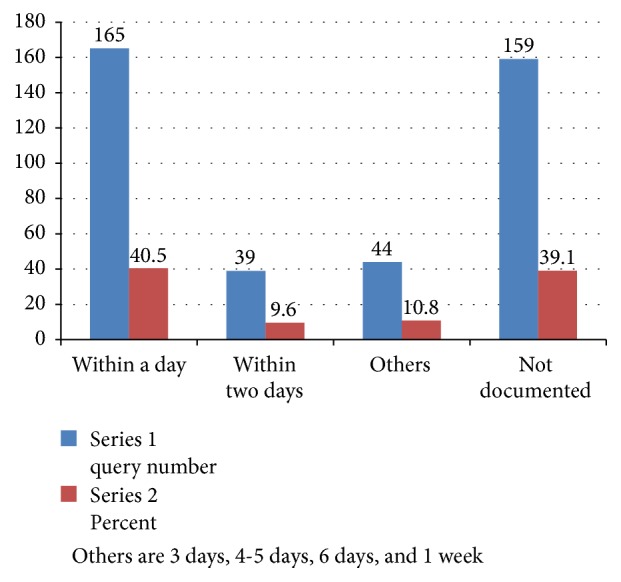
Time frame for responding to the queries to drug information centers during the study period.

Although for a significant amount of queries (n=159) the time frame for reply was not documented (39.1 %), 40.5 % of the replies were given within 24 hours of receiving, 9.6 % within 48 hours, and the rest 10.8 % within 3 days, 4-5 days, 6 days, and even a week (Figure 3).

### 3.6. Facilities Available in the DICs

The DICs in the five centers are having appropriate utensils and resources as shown in Table 5 to carry out their duties. Staff number varies from having a single pharmacist to four pharmacists per center. Additional staff other than pharmacists like computer specialists are present in some of the centers.

All the studied DICs work for 8 hours per day according to the normal public institutional working hour's norm in Ethiopia. Among the assessed DICs, TASH is the only center involved in communication with other DICs, namely, with the DICs of Gondar University, Bonga Health Center, St. Paulos Hospital, Mekelle University, Jimma University, and Ras Desta Hospital, where both are health care institutes located in different parts of the country.

### 3.7. Working Area and Some Accessories

The DIC directors claimed that the working area was suitably built in three of the DICs while it is not the case with the rest. The reasons forwarded by one were unreachable location of the DIC, poor Internet access, lack of tools to provide the service, inadequate number of books, and absence of databases and journals, while the other one mentioned that lack of up to date textbooks in addition to inaccessible location of the DIC is hindering the service.

### 3.8. Information Search Strategies

Four of the DICs mentioned that they had utilized all relevant references important to respond to the queries. However, one DIC used only primary literature. In addition, two DICs had consulted advisors necessary to respond to a certain query. During literature evaluation, all of the DICs except one (St. Peter) used various data sources instead of a single source. However, it is the responsibility of the drug information pharmacist to analyze resources and address the queries in all of the DICs. In three of the DICs full details about references were offered when responding to queries. In one DIC reasons had been stated in case when literature provided was found to be inadequate. In addition, animal and/or***in vitro*** data were used for the response. Personal knowledge was substantiated by literature whenever it was important.

### 3.9. Background Information Gathering

The DICs collect necessary background information when queries were submitted. All of the DICs received almost full demographics from the requesters. The information included consisted of full name and location of the inquirer, address and profession of the inquirer, category of the query, and the drug's dosage form. Four of the DICs gather and document date and time of received queries with the time frame to reply, context of the query, current disease history, medication history, and history of complication. They also confirm with the enquirer that the query is understood. But reason for query submission was only recorded in two of the DICs.

### 3.10. Documentation

Four of them used file folders for documentation while one of the four also used log books as a plus. Documentation method was not stated in one of the DIC.

### 3.11. Challenges

The challenges faced by each DIC were different. TASH DIC was challenged by less/not enough awareness done to the health care professionals, low budget to purchase some resources to facilitate the service, and availability of only outdated textbooks, while, in St. Peter and Mekelle, the challenges mentioned include poor Internet access, lack of photocopier and printer, not enough books, and no database and journals. The Jimma DIC had drawbacks of nonavailability of working materials necessary for the DIC service and shortage of skilled or trained staff that fits the DIC position. At Gondar DIC there was a challenge of lack of Internet access and poor advertisement of its function. A recommendation by all of them was for the DIC service to be improved, and the problems specific to each DIC should be addressed.

## 4. Discussion

This study is a pioneer, apart from the previous assessment done in Addis Ababa by the first author and his team [18]. It attempts to assess the different functions in the DICs of the teaching university hospitals of Ethiopia. The demand for drug information is observed not only by health care professionals but also among general public including patients. It was found that there were 407 documented queries that were included in the study. The DICs assessed are the four university hospitals and one public hospital in Addis Ababa.

The DIC found in Ayder Referral Hospital, that became functional in 2013, contributed greater number of queries eligible for this study. This DIC handled near 11 queries per month. But compared to Uganda and Nepal which received 27 and 28 queries per month, respectively, this number is low [16, 25]. The reason for the Mekelle DIC to have higher number of queries is because the setup there is designed to engage graduating pharmacy students. The students actually participate in obtaining or collecting queries from different clinical wards. They respond and document the queries as part of their attachment to the DIC. These activities highly increased the query numbers that would normally be forwarded to the center. Such practice is not evident in TASH. At TASH only directly forwarded questions are documented and students are given the queries at the DIC query pool. Hence this may be adopted in other DIC centers in Ethiopia or elsewhere to increase the number of queries to be submitted to each DIC. Although a previous need assessment study in Ethiopia at Jimma and its environs asserted the high demand for the DI resources and services [27], the number of queries submitted and documented to each DIC is very low. One factor that had contributed to this might be not documenting all the queries. This is the usual case in oral queries during ward rounds. A Saudi study also confirmed underutilization of DICs there too in comparison to the developed world like the USA and UK [28].

Most of the queries (60 %) were from public hospitals. This may be due to distance between the center and the health care facilities [18]. DICs were located within the hospitals, as it was also observed that the inquirers that held the highest percentage in each DIC were from the hospitals themselves.

The receipt of some queries from other facilities referred to as others which include Community Pharmacy, Pharmaceutical Companies, and Private Hospital. This could be an indication that the number of users is likely to increase with more promotional activities on the existence and functioning of the DICs. Health care workers account for the highest (99.5 %) number of inquiries, although the public used the centers. This explains the need for accurate, unbiased drug information. Unlike the previous study [18] in Addis Ababa where physicians were the major drug information requesters, this time pharmacists happened to be taking the lead. This might be due to the implementation of clinical pharmacy services in these hospitals after the clinically oriented and MSc in clinical pharmacy recent graduates are joining the inpatient ward pharmacy services [29]. Indian studies found also physicians and postgraduate students to be the major users of the DICs [30, 31].

Doctors and nurses are accepting pharmacists as having more knowledge about drugs than themselves thus requesting drug information service from them.

The public submitted very small inquiries to the centers. This result was in accordance with other researches [16, 32]. But in Iran patients and their relatives were found to be major users of a drug and poison information center [33]. Limited utilization of the DICs in Ethiopia by the public might be due to less awareness. But patients are highly in need of accurate drug information service for better treatment outcome. A research in south Indian teaching hospital documented the order of usage of the DIC as students, nurses, and physicians from the highest to lowest user, respectively [7]. However this study had demonstrated that the highest frequency of use is coming from pharmacists themselves (63.5%), followed by health care students (12.7 %), and physicians (8.4 %). Other studies in Uganda and India reported doctors being the highest users [16, 30]. This could be because physicians are the main prescribers and thus need to access appropriate drug information. But in Ethiopia in this study unlike the previous ones [18], pharmacists were found to be the highest DIC service users which might be attributed to the pharmacists participation in clinical pharmacy services as well as more awareness to it. In Brazilian study pharmacists were the second highest requesters next to nurses to utilize the DIC services while medical teams (where physicians are members) are least inquirers [34]. Some of the professionals categorized as others include health officer student, health officer, patient, laboratory technician, and microbiologist.

The majority of the mode of receiving query was walk-in (45.4 %), although quite a good number of the queries did not document the mode of receiving (37.8 %). However, when comparing the mode of receiving queries that had utilized telephone (5.0 %) and morning round (9.8 %), more inquirers did it in person due to easy access. Moreover as a developing country institution, free institutional telephone lines might not be adequately available to the professionals in the hospitals. There is no also cost covering scheme to use private cell phones. The trend is similar to the 2011 study in India but opposite to an Iranian study where all queries were submitted by telephone [33]. This mode will enable the requester to get in-depth information. This might be true for this study too. It can also be concluded that for one of the DICs in this study, namely, TASH, though the center is not so close to the clinical setting, pharmacists are dedicated to forward their questions while walking in to the center to read books and search for different data using the Internet connection and databases in the center. The other reason where telephone is less widely used might be due to lack of documentation.

The main concern of the queries is for better treatment outcome to the patient. 34.3 % of the inquiries address such issues but still 39.3 % were found to be not documented. 25. 4 % and 0.5 % queries were submitted to get new knowledge and education/academic purposes, respectively. This is however not in line with an Indian study in 2012 that puts the number of inquiries which asked for the purpose of updating knowledge (56.55 %) followed by for better patient treatment (31.14 %) and 1.65 % for academic purposes [4].

The most important areas of information identified were drug interaction (19.7 %), therapeutic use (17.8 %), general product information (13.7 %), product/dosage form availability (6.7 %), adverse effect (8.7%), and others (20.4 %). In most studies, including the one in Uganda, queries on therapy were reported to be the most common. In the current study similar results were observed (17.8 %). Therefore, this emphasizes the likely role of the DICs in improving the quality of patient care [16]. Other areas of information offered include product identification, dosage recommendation for adult, pediatric, geriatric cases, compounding, method/route of administration, drugs in pregnancy and lactation, contraceptives, pharmacology, pharmacokinetics, product availability, compatibility/stability, price, dietary supplements, local/foreign drug equivalence, diagnosis, side effect, contraindication, duration of treatment, dosage form, overdose, Ethiopian traditional medicine, drug food interaction, pathophysiology, drug of choice, pharmaceutical information, dose calculation, renal dose adjustment, therapeutic failure, treatment failure, dose, addiction, toxicology, and antidote.

Antimicrobial class of drugs (23.3 %) accounts for the highest inquiries in this study. This is in line with previous studies (22% - 56 %) [16, 32]. Antimicrobials were reported as second highest next to antidepressants group in an assessment done in Iran [33]. This is in line with the prevalence of infectious diseases in the country compared to other types of diseases [35]. The next drug class which is prevalent is antihypertensives (11.4 %). It informs us also the rise in the epidemiology of chronic diseases like hypertension in the country [36]. The predominance of pharmacological classes categorized under others (32.1 %) is because of long lists of drug classes which sums up but individually their frequency being insignificant.

An Indian study described that, for the purpose of addressing inquiries that received both, primary, secondary, and tertiary references were used. The percent frequency of the use is Micromedex (52.45 %), text books (22.38 %), and web sites (10.49 %) [30]. In Iran too, Micromedex was used as the most common reference accounting to 72 % [33]. In this study web sites (31.4 %) were commonly used followed by Micromedex (19 %), UpToDate (15.5 %), text books (15.4 %), and journals (14.3 %). Thus availability of a fast Internet connection will affect DIC services. More researches had also mentioned that Micromedex was the common DI reference and described as being the best in responding to inquiries within 30 minutes [7, 17, 33]. Electronic resources including Micromedex are absent at Jimma DIC although it offers comparative advantage over text books. Web sites were commonly used because it is easy to get information from them. Primary sources like journals were less frequently used. The reasons might be easiness of the queries to answer by tertiary references, unavailability, or rare availability of primary references and DI pharmacists' level of understanding of such materials [37]. Information from guidelines, Drugdex, leaflet, phone contact, protocols, store man, and monograph was also used to reply queries. They were among the references categorized as others.

DICs are expected to answer the requests within three days. In this study 39.1 % of the responses did not mention the time it took to reply. However 40.5 % responded within a day. When this result is compared to other studies, it is above 30.15% and lower than 68.03% addressed in a day from 335 and 122 queries, respectively, in studies elsewhere [3, 4]. The sole most parameter that predicts the time duration for a response is the type of reference used. Tertiary sources usually reduce the time needed to respond to a query [16]. Previous studies had already demonstrated that, among electronic sources, the use of databases (Drugdex, lexi-drugs) help to address the queries in shorter period compared to Internet. Drugdex a part and parcel of Micromedex is the second common reference used in this research. This may be due to easy to use arrangement of this database [38]. More than half (51.1 %) of the queries were addressed within two days.

Replies were made according to the preferred method by the enquirer. 79.36 % of the 407 queries were replied in writing. When this value is compared to a 2012 study in India (27.87 %), 46.2 % of the reply was with reference literature which is lower than another research (63.11 %) [4]. But it is very highly compared to the previous study in Ethiopia [18]. The reason for such case may be due to absence of documenting or less use of primary references. On the other hand those queries that were responded orally account for 11.05 %, a slightly greater value from the Indian study (9.02 %). The main purpose of many queries was better care which requires fast response to patients thus the need for immediate oral reply. This is in line with investigations done in India [7, 39]. 7.8 % of the replies were done by phone. As Ethiopian telecommunication infrastructure had been rapidly expanded, growing use of it for DIC services is highly anticipated. Such a center has been reported to be successful in Nepal [40]. DI personnel had mentioned that rephrased printouts were supplied after quick replies through the phone.

For most of the results obtained above poor documentation was an issue. The basic elements listed by the Pan African Health Organization (PAHO) and other additional elements are the consecutive number of inquiry, demographics of inquirer, type of inquiry, question and answer, references used, the type of answer (oral or written), contact information, institution of origin, and person answering the inquiry. Date and time of inquiry and response, key words, purpose of query, data on the patient, and mode of receiving queries were the least documented ones in the DICs of the public hospitals [1, 8]. This problem with documentation could be due to the lack of professional attitude towards the job since there is lack of staff and lack of incentives or training. Poor documentation and dissemination of the little available information may lead to the lack of adequate drug information service [40].

The result for the facilities (Table 5) was a current observation. According to the directors, most of the DICs fulfilled the requirements of facilities set by the Ethiopian Food Medicines Health Care Administration and Control Authority (EFMHACA). Regarding space for the provision of the services, each had their own office with adequate size and furniture which included desks, chairs, and shelves. Communication tools like Internet and telephones were available. Computers, printers, reference materials like books, and electronic information resources which are important in maintaining the services were present in most of the centers.

The challenges and drawbacks of the centers that mostly occurred are similar to the ones faced in developing countries [41]. These can be viewed in three aspects: inquirers themselves, resources, and professionals available. According to the heads of the centers, physicians and other health care workers unawareness to the DIC services was mentioned as a drawback. However a study in Malaysia [32] had demonstrated that promotional activities did not last long. Thus for promotional activities to create long lasting impressions, they should be done frequently. It is the role of both doctors and pharmacists to do awareness and promotion of DIC services [16]. The DIC pharmacists' professional capacity is also very important for efficient drug information service [42].

The centers suffer from the lack of databases as well as recent edition text books to be used as references. The pharmacists working in the centers usually participate also in other pharmaceutical activities including teaching [41]. A way to contact DI pharmacists after working hours was absent. However this can easily be done by posting contact mechanisms like telephone and email address on the doors. But some employ web based submission of queries.

DICs in Ethiopia are involved in making and disseminating brochures and newsletters for teaching purpose about drug information of importance. This and other services of DICs in Ethiopia are similar to other countries [8, 43, 44]. Elsewhere in other countries (Argentina, Costa Rica, Italy, and Iran), DICs strongly participate in course development and offering to students and involve different committees in the hospital. Similarly as most of the DICs in this study are housed in university teaching hospitals, they actively participate as preceptors and sites for pharmacy students in the DIC service training. However, academic staff in the university rather than the DIC pharmacists perform the lectures, researches, and curricular development of the courses concerning DIC services in Ethiopia.

### 4.1. Limitations

This research solely depended on queries submitted and documented in the DICs. However standard formats are absent to document the queries and responses in the centers. There is also leniency to document all of the received and responded queries (especially for those oral communications done) which might down play the overall load and work out put in the assessed DICs.

## 5. Conclusion

The assessment had shown that drug information queries in Ethiopia deal mainly with pharmacotherapy, side effect, product stock in/out, general characteristics, pharmacokinetics, and dynamics about antimicrobials. The resources used as references for drug information were web sites, Micromedex, and UpToDate™. DICs in most health care institutes were permanently staffed by pharmacists. All the centers are established with optimal rooms and basic equipment, although more materials and inputs are required in improving their services.

## Figures and Tables

**Table 1:** Queries, documentation period, requester characteristics, and receiving modes for the drug information centers of the hospitals.

**Hospital**	**Documented queries (No.)**	**Eligible for analysis (No.)**	**Percentage (**%**)**	**Documentation period**
TASH	116	116	28.5	November/2013-August/2015 (22 months)
St. Peter	11	11	2.7	Not recorded
Mekelle	143	143	35.1	July/2013-August/2014 (14 months)
Jimma	57	57	14.0	July/2012-July/2015 ( 36 months)
Gondar	112	80	19.7	January/2013-May/2015 (29 months)

**Total**	**439**	**407**	**100**	

**Drug information requesters**				
Health Professionals		385	99.5%	
General Public		2	0.5%	

Total		387	100.0%	

Missing		20	-* *-* *-* *-	
**Mode of receiving query**				
	No.	%		
Morning round	39	9.8%		
Phone	20	5.0%		
In person (Walk in)	181	45.4%		
Other*∗*	8	2.0%		
Not documented	151	37.8%		

Total	399+8	100.0%		

Missing/	8	-* *-* *-* *-		

Others are email and DIC web sites.

**Table 2:** Types and purpose of queries received.

**Types**		**Responses**	
		**N**	**Percent**
Type of queries	General product information	80	13.7%
	Adverse Effect	51	8.7%
	Availability of Dosage forms	39	6.7%
	Drug interaction	115	19.7%
	Therapeutic use	104	17.8%
	Others	119	20.4%
	Not documented	75	12.9%

**Total**		**583**	**100.0**%

Purpose			Percent

Valid	Better patient care	138	33.9
	Update the knowledge	102	25.1
	Education/academic	2	.5
	Not documented	160	39.3
	Total	402	98.8
Missing		5	1.2

**Total**		**407**	**100.0**

Others include product identification, adult dosage recommendation, pediatric dosage recommendation, geriatric dosage recommendation, compounding, method/rate of administration, drugs in pregnancy and lactation, contraceptives, pharmacology, pharmacokinetics, product availability, compatibility/stability, price, dietary supplements, local/foreign drug equivalence, diagnosis, side effect, contraindication, duration of treatment, dosage form, overdose, Ethiopian traditional medicine, drug food interaction, pathophysiology, drug of choice, pharmaceutical information, dose calculation, renal dose adjustment, therapeutic failure, treatment failure, dose, addiction, toxicology, and antidote.

**Table 3:** Pharmacological class among N=400 queries that contain well-documented pharmacological class identifiable drugs while N= 7 of them miss such information.

Pharmacology		Responses	
		N	Percent
	Antibiotics	125	23.3%
	Antiretroviral	28	5.2%
	Antipain	46	8.6%
	Antihelmintic	35	6.5%
	Antihypertensive	61	11.4%
	Other	172	32.1%
	Not applicable	69	12.9%

Total		536	100.0%

Others include anticoagulant, anti-inflammatory, antiplatelet, antiacid, anticancer, anticonvulsant, antifungal, antituberculosis, antiallergy, antidepressant, antimalarial, antiemetic, antiviral, antipsychotic, antiasthmatic, antihypoglycemic, antidiabetic, antianxiety, adsorbent, anesthetic, inotropic agents, hormonal drugs, contraceptives, erectile dysfunction, congestive heart failure, food supplement, vitamin, and mineral supplements.

**Table 4:** References used and mode of reply to queries.

**Reference**	**No.**	%
**Valid (documented)**	259	63.6 %
**Missing (not documented)**	148	36.4 %
Textbooks	88	15.4 %
Micromedex	109	19.0 %
Websites	180	31.4 %
Journals	82	14.3 %
UpToDate	89	15.5 %
Others^a^	18	3.1 %
Not documented	7	1.2 %

**Total**	573	100.0 %

**Mode of Reply**		

Valid (documented)	357	87.7 %
Missing (not documented)	50	12.3 %
written	135	41.8 %
Rephrased Printout	188	58.2 %

Total	323	100.0 %

Verbal		
Phone	32	71.1 %
In person	13	28.9 %

**Total**	45	100.0 %

^a^Others include guidelines, Drugdex, leaflet, phone contact, protocols, store man, monograph.

**Table 5:** Characteristics of the drug information centers in the studied hospitals.

**Utensils**	**Hospitals**				
	**TASH**	**St. Peter**	**Mekelle**	**Jimma**	**Gondar**
Room Area	24.4 m^2^	5.2 m^2^	45 m^2^	13 m^2^	22 m^2^
Computers	✓ (5)	✓	✓ (6)	x	✓ (12)
Printers	✓ (3)	x	x	✓ (1)	✓ (1)
Photocopier	✓ (1)	X	x	x	x
Textbooks	✓ (Plenty)	✓ (30)	✓ (20)	x	✓ (Plenty)
Journals	✓ (Plenty)	x	x	x	X
Electronic information Resources	✓ (1)	X	✓ (1)	x	✓ (1)
Telephone	✓	X	x	x	X
Internet	✓	✓	✓	x	x
Pharmacist staff	2	1	3	1	4

✓: present; x: absent.

## Data Availability

The data used to support the findings of this study are included within the article.

## Abbreviations

DIC: Drug information center
DIS: Drug information service
DI: Drug information
EFMHACA: Ethiopian Food Medicine Health Care Administration and Control Authority
PFSA: Pharmaceutical Fund and Supply Agency of Ethiopia
TASH: Tikur Anbessa Specialized Hospital
PAHO: Pan African Health Organization
CDC: Centers for Disease Control and Prevention, Atlanta, Georgia
SPSS: Statistical Package for Social Sciences
TB: Tuberculosis
MCH: Maternal and child health
PEPFAR: The United States President's Emergency Plan for AIDS Relief.

## Additional Points

(i) Drug information centers can be established and their services can be maintained in a developing country setting. (ii) Lack of facilities in terms of Internet connections and databases limits drug information services. (iii) Queries are related to the prevailing main diseases and activities of the hospitals in which the DICs are operational. (iv) Drug information requests are related to medicines pharmacotherapy, its stock out/in, and side effects. The pharmacological class commonly submitted in the queries concerns antimicrobials.
